# A Non-linear Relationship Between Selective Attention and Associated ERP Markers Across the Lifespan

**DOI:** 10.3389/fpsyg.2019.00030

**Published:** 2019-01-28

**Authors:** Eva-Maria Reuter, Solveig Vieluf, Flora Koutsandreou, Lena Hübner, Henning Budde, Ben Godde, Claudia Voelcker-Rehage

**Affiliations:** ^1^Centre for Sensorimotor Performance, School of Human Movement and Nutrition Sciences, The University of Queensland, Brisbane, QLD, Australia; ^2^Institute of Sports Medicine, Paderborn University, Paderborn, Germany; ^3^Faculty of Human Sciences, Medical School Hamburg, Hamburg, Germany; ^4^Institute of Human Movement Science and Health, Chemnitz University of Technology, Chemnitz, Germany; ^5^Physical Activity, Physical Education, Health and Sport Research Centre, Sports Science Department, School of Science and Engineering, Reykjavik University, Reykjavik, Iceland; ^6^Institute of Sport Science and Innovations, Lithuanian Sports University, Kaunas, Lithuania; ^7^Department of Psychology and Methods, Jacobs University, Bremen, Germany

**Keywords:** EEG/ERP, aging, inhibition, Flanker, development, brain

## Abstract

The ability to selectively attend to task-relevant information increases throughout childhood and decreases in older age. Here, we intended to investigate these opposing developmental trajectories, to assess whether gains and losses early and late in life are associated with similar or different electrophysiological changes, and to get a better understanding about the development in middle-adulthood. We (re-)analyzed behavioral and electrophysiological data of 211 participants, who performed a colored Flanker task while their Electroencephalography (EEG) was recorded. Participants were subdivided into six groups depending on their age, ranging from 8 to 83 years. We analyzed response speed and accuracy as well as the event replated potential (ERP) components P1 and N1, associated with visual processing and attention, N2 as marker of interference suppression and cognitive control, and P3 as a marker of cognitive updating and stimulus categorization. Response speed and accuracy were low early and later in life, with peak performance in young adults. Similarly, ERP latencies of all components and P1 and N1 amplitudes followed a u-shape pattern with shortest latencies and smallest amplitudes occurring in middle-age. N2 amplitudes were larger in children, and for incongruent stimuli in adults middle-aged and older. P3 amplitudes showed a parietal-to-frontal shift with age. Further, group-wise regression analyses suggested that children’s performance depended on cognitive processing speed, while older adults’ performance depended on cognitive resources. Together these results imply that different mechanisms restrict performance early and late in life and suggest a non-linear relationship between electrophysiological markers and performance in the Flanker task across the lifespan.

## Introduction

Selective attention allows to successfully focus on goal relevant information while inhibiting irrelevant information. As such selective attention is part of inhibitory control [see Diamond (2013) for taxonomy and detailed review on inhibitory control] and is assumed to be an important marker of general cognitive functioning, behavioral–emotional control, and academic attainment (Treitz et al., 2007; Healey et al., 2008; Downes et al., 2017; Moore and Zirnsak, 2017). Along with other executive functions, selective attention grows throughout childhood and adolescences and declines in older age. While children increase their attentional capacity due to practice and (brain) maturation, in older ages performance levels are assumed to decrease due to declines in processing capacities (Mueller et al., 2008). Such, inverse developmental changes at both ends of the lifespan can be observed on behavioral and neurophysiological levels and allow to infer about progressive and regressive changes of brain functioning. Importantly however, lower levels of behavioral performance in children and older adults might have different underlying mechanism, suggesting a non-linear relationship between neurophysiological markers and cognitive performance across the lifespan (Hommel et al., 2004). Here, by combining data from several studies, we aimed to investigate selective attention in a large sample across the lifespan. Importantly, we did not only measure behavioral, but also electrophysiological markers of selective attention, which allows us to observe lifespan trajectories and to identify neural makers associated with growth and losses.

Attentional control develops throughout childhood and adolescence until young adulthood, in parallel with the development of the prefrontal cortex (Sowell et al., 2003; Steinberg, 2005; Lenroot and Giedd, 2006) and the increasing differentiation of brain networks (Anokhin et al., 1996, 2000). Previous research evidenced that children (approximately <9 years of age) less effectively attend to relevant stimuli than young adults (Ridderinkhof et al., 1997; Davies et al., 2004; Rueda et al., 2004; Davidson et al., 2006; Waszak et al., 2010). Specifically, they require more time to identify target stimuli and their behavior is relatively more error prone, especially when targets are surrounded by distracting, irrelevant stimuli. In older adults, brain networks dedifferentiate and the brain undergoes functional and structural changes (Rajah and D’Esposito, 2005; Fjell and Walhovd, 2010; Carp et al., 2011), while selective attention declines. Similarly to children, older adults are less able to focus attention on task-relevant information and to inhibit task-irrelevant information from simultaneous and competing information streams (Hasher and Zacks, 1988; Treitz et al., 2007; Wild-Wall et al., 2008; Korsch et al., 2014; Reuter et al., 2017). This age-related decline is likely to start in middle-age with an even steeper decline in older ages. However, considerable fewer research has been devoted to the middle-aged lifespan (Cid-Fernández et al., 2014). Notably, age-related changes become not always evident on a behavioral level due to neural compensation (Reuter-Lorenz and Park, 2014). Hence, differences between age groups might only be reflected in changed neural activity. In addition, age-related changes in the brain are not necessarily mirror-symmetrical at both ends of the lifespan (Span et al., 2004). Together, this highlights the benefit of including neuronal makers, when studying lifespan development of selective attention.

On a neurophysiological level, age-related changes are evidenced, for instance, in changes in the latency, amplitude, and distribution of the electroencephalographic response to sensory stimuli (Mueller et al., 2008). Specifically, P1 and N1, early event-related potential (ERP) markers of sensory encoding and processing and components sensitive to endogenous attention to sensory stimuli features (Posner, 1980), are larger and have longer latencies in children (Taylor and Khan, 2000; Itier and Taylor, 2004; Tomé et al., 2015) and older adults (Anderer et al., 1996). These age-related changes are thought to reflect deficits in engaging specific inhibitory control, and reduced processing speed, respectively (Anguera and Gazzaley, 2012). In addition, age-related changes in scalp-to-skull conductivity ratio (for discussion, see Wendel et al., 2010), as well as age-related compensatory engagement of visual attentional mechanism (Wild-Wall et al., 2008) might play a role.

Also subsequent ERP components N2, associated with interference suppression and cognitive control, and P3, associated with updating, categorization, and allocation of attentional resources, differ in size, latency, and distribution across the lifespan (Downes et al., 2017). N2 and P3 latencies are longer in children (Rozhkov et al., 2009; Tsai et al., 2012) and older adults (van Dinteren et al., 2014b) reflecting changes in neural speed and efficiency. In contrast, N2 amplitudes seem to change unidirectional with age. N2 amplitudes reduce from childhood to young adulthood (Johnstone et al., 1996; Mueller et al., 2008; Downes et al., 2017), and decrease further from adulthood to old age (Kropotov et al., 2016). P3 amplitudes increase from early childhood to late childhood to an adult level in children at about 12 years of age (for overview and discussion, see Mueller et al., 2008; van Dinteren et al., 2014a) and decrease in older age (Polich, 1997; Friedman, 2012).

In addition, across the lifespan, the distribution of P3 changes: while children (Taylor, 1988; Johnstone et al., 1996) and young adults typically reveal a strong parietal P3 focus, the P3 is more equipotent between frontal and parietal electrode sides with increasing age, or is even larger over frontal than parietal electrodes in older adults (Pfefferbaum et al., 1984; Polich, 1997; Reuter et al., 2013, 2017). However, N2 and P3 amplitudes are modulated less by task demands in both children and elderly. Specifically, while young adults selectively show larger N2 and P3 components to stimuli requiring more attentional resources (e.g., incongruent (IC), unexpected, or target stimuli, compared to standard or non-target stimuli), children and older adults’ N2 and P3 amplitudes are less differentiated, reflecting a reduced ability to control attentional focus (Johnstone et al., 1996; Mueller et al., 2008; Boucher et al., 2010; Anguera and Gazzaley, 2012).

In sum, it seems that behavioral measures of selective attention and some Electroencephalography (EEG) markers (i.e., ERP latencies and P1 and N1 amplitudes) follow a u-shape pattern across the lifespan, while other EEG makers (i.e., N2 amplitudes and P3 amplitudes and distribution) differ between children and older adults. Moreover, while behavior and ERPs have been compared exhaustively between different age groups, little is known about how such EEG markers can predict behavioral outcomes across the lifespan. Therefore, here we aimed to reveal, whether behavioral deficits in children and older adults are associated with similar or different EEG responses. We intended to identify symmetrical trajectories in childhood and old age and to distinguish those from different neuronal processes at both poles of the lifespan. Furthermore, in contrast to the majority of earlier studies addressing lifespan changes in selective attention, our data set also includes the middle-age lifespan, largely underrepresented in developmental research. This allows us to infer the age of peak performance and functioning and to discuss the development of age-related change across the lifespan. We combined data from three studies in which we collected EEG and behavioral measures in participants aged 8–83 years performing a colored Flanker task (Eriksen and Eriksen, 1974). Participants were asked to respond to a target stimulus while ignoring non-target stimuli surrounding the target. We analyzed response times (RT) and accuracy as well as ERP markers of visual processing (specifically visual P1 and N1), and of cognitive processing (specifically N2 and P3). We expected to reveal a u-shaped relation between age and behavioral performance in the flanker task with both children and older adults perform below the level of young and middle-aged adults. We also expected to confirm age-related changes in ERP markers. Most interestingly, we aimed to explore, whether the same or different EEG markers predict performance in different age groups and thus to infer about the underlying mechanism which might determine performance limits at both ends of the lifespan.

## Materials and Methods

### Participants

Data (*n* = 222) for this study were collapsed between three experimental studies. Ninety-two data sets were collected as part of the BremenHandStudy@Jacobs, referred to as Study 1 (age range 20–81 years, 54 females). These participants were recruited through announcements in local newspapers or were part of the participant pool of Jacobs University Bremen, Germany. Participants received 8 €/h for their participation (for details, see Winneke et al., 2012; Reuter et al., 2017). Eighty-one data sets were collected as part of the re-LOAD project, referred to as Study 2 (age range 20–83 years, all female). Data collection was embedded within a project on motor learning in older adults (for details, see also Hübner et al., 2018a,b). Older participants were re-recruited from a previous study of our research group at Jacobs University Bremen (all agreed to store their contact data in a participant database). Young adults were recruited with flyers and mailing lists from Jacobs University Bremen and University of Bremen. All adult participants in Studies 1 and 2 gave their written informed consent to take part in the study. Forty-nine data sets were collected as part of the CEBRA project, referred to as Study 3 (age range 8–11 years, 25 females; Koutsandreou et al., 2016). Data for Study 3 were collected in three different primary schools in Paderborn, Germany, as part of a study on physical and mental fitness in primary school children. Guardians provided written informed consent. Ethical approval for Studies 1 and 3 was granted by the German Psychological Society, and for Study 2 by the Ethics Committee of the Faculty of Humanities of the Saarland University, Germany. The results of 84 data sets of the current sample were previously published with a focus on a different research question (i.e., the influence of physical activity on executive control in middle-aged adults, 44 data sets, Winneke et al., 2012; and the role of the parietal-to-frontal shift with regard to executive control in participants 75 years and older, 40 data sets; Reuter et al., 2017). Thus, the current data set consists of data that have not been published previously as well as published data (see Table 1 for details).

**Table 1:** Overview of experimental groups.

Final sample in current study	Children (*n* = 46, 24 females)	Adults young (*n* = 39, 34 females)	Adults early middle-aged (*n* = 21, 12 females)	Adults late middle-aged (*n* = 25, 14 females)	Adults older < 75 years (*n* = 40, 36 females)	Adults older > 75 years (*n* = 40, 31 females)
Data sets included in previous reports	0	14^∗^	20^#^	24^#^	0	26^∗^
Number of data sets recorded per study	Study 3 (*n* = 46)	Study 1 (*n* = 14) Study 2 (*n* = 25)	Study 1 (*n* = 21)	Study 1 (*n* = 25)	Study 2 (*n* = 40)	Study 1 (*n* = 26) Study 2 (*n* = 14)
Age (years)	9.32 (0.65)	22.85 (2.50)	42.62 (3.61)	59.04 (2.39)	71.93 (3.04)	78.16 (1.98)

*Standard deviations are given in parenthesis. ^∗^, Reported in Reuter et al. (2017); ^#^, reported in Winneke et al. (2012); all previously recorded data sets were collected as part of Study 1.*

In participants older than 65 years we assessed cognitive functioning using the Mini Mental State Examination (MMSE; Folstein et al., 1975) (Study 1) or the Montreal Cognitive Assessment (MoCA; Nasreddine et al., 2005) (Study 2) to exclude participants with cognitive impairments or dementia. All participants scored higher than 27 points in the MMSE or at least 23 points in the MoCA, indicating normal cognitive functioning (Julayanont and Nasreddine, 2017).

In sum, 222 data sets were analyzed initially (8–83 years of age; 160 females). We excluded five participants due to overall poor EEG data quality and six additional participants as they did not meet the criteria of having reached a minimum of 35 artifact-free EEG trials with correct behavioral response in at least one of the conditions. This final sample consisted of 211 healthy participants (8–83 years of age; 151 females). This sample was divided into subgroups depending on age. The following age categories were formed: Children, 8–10 years of age; adults young, 20–29 years of age; adults early middle-aged, 36–48 years of age; adults late middle-aged, 55–64 years of age; adults old < 75, 66–75 years of age; adults old > 75, 76–83 years of age (see Table 1 for sample characteristics). Age categories were formed based on availability of data set from the three studies, so that none of the groups comprises an age range of >15 years and following (Newman and Newman, 1975). Note that this allowed us to extend the commonly studied groups of young adults in comparison to children, and/or older adults (typically 65–75 years of age) by three additional groups. Two groups comprising the widely under-researched middle-aged age range and one group of the very old (>75 years of age).

### Experimental Task and Procedure

All data were recorded with the same recording equipment and data of Studies 1 and 2, were collected in the same laboratory set up at the Jacobs University Bremen. Data of Study 3 were recorded at primary schools in Paderborn. Participants performed a color Flanker task under three conditions: C, IC, and neutral (N). Color flanker tasks have been successfully employed in children (McDermott et al., 2007) and older adults (McDermott et al., 2007; Voelcker-Rehage et al., 2010; Waszak et al., 2010). The task employed here was adapted from Li et al. (2004) and has been previously described in Winneke et al. (2012) and Reuter et al. (2017). In brief, stimuli consisted of one central and four surrounding colored discs. The central disc was the target and was either green or red. The task was to press a button indicating if the target was green or red using the index and middle finger of the right hand, respectively. The surrounding discs were either (a) the same color as the target disc (C condition), (b) the opposite color (e.g., red/green flankers with a green/red target; IC condition), or (c) a N blue color, which was not associated with any response (N condition). Testing protocols and procedures were identical for all data recorded between studies, with the following exceptions. In Studies 1 and 3, 300 trials (approximately 100 trials per condition) were performed while in Study 2 only 150 trials (approximately 50 trials per condition) were performed. The order of conditions was randomized. The number of trials was reduced for that study, due to an extensive test battery on other motor and cognitive performance measures for participants. Further, in Studies 1 and 3, stimuli were presented for 200 ms, in Study 2 stimuli were presented for 500 ms. In all studies, stimuli were preceded by a fixation cross (300 ms) and a blank screen (200 ms) and succeeded by a variable intertrial interval of about 950 ms; range 800–1100 ms).

Participants were instructed to respond as quickly and accurately as possible. They conducted 20 practice trials prior to the experiment. For the current study, we focused on the C and IC condition. We analyzed response accuracy, i.e., the percentage of correct responses, and the median RT for the trials in which a correct response was made between 100 and 1200 ms after stimulus onset. Following Niemann et al. (2014), in order to offset speed against accuracy, a standardized performance index (*q*-score) was calculated based on median RT and response accuracy measures per condition: *q*-score = RT correct responses/IQ standardized percentage of correct responses. IQ standardization standardizes the data to a normal model, with a mean of 100 and a standard deviation of 15. A lower *q*-score represents faster, more accurate performance, on the test.

### EEG Data Recording and ERP Analysis

EEG data were recorded using the same 32-channel active electrode system (ActiveTwo, BioSemi, Amsterdam, Netherlands) in all three studies. Electrodes were placed according to the 10–20 system (Jasper, 1958). Vertical and horizontal eye movements as well as mastoid potentials were recorded with six facial electrodes designed for body-surface applications. The signal was digitized with a sampling rate of 2048 Hz and online band pass filtered between 0.16 and 100 Hz. Offline analyses of the EEG data were performed using Brain Vision Analyzer Software 2.0 (Brain Products, Munich, Germany). For ERP analyses, the signal was offline down-sampled to 512 Hz and re-referenced to linked mastoids. A low-pass filter of 30 Hz and a notch filter of 50 Hz were applied. The data were then segmented into 900 ms segments of 100 until 800 ms from stimulus onset, and baseline corrected relative to the 100 ms period prior to stimulus onset. Blinks were corrected using ocular artifact removal based on an algorithm (Gratton et al., 1983) implemented in the analysis software.

Trials with voltage differences of more than 100 μV within 100 ms were automatically detected and rejected as artifacts. Only trials in which participants made correct responses between 100 and 1200 ms after stimulus onset were analyzed. ERP data were obtained by averaged segments and we analyzed P1 and N1 as markers of visual processing and visual attention, N2 as marker for interference suppression and cognitive control, and P3, associated with updating, categorization, and allocation of attentional resources. Inspection of grand-average data across the different subsamples revealed P1 and N1 peaking at about 75 and 160 ms, respectively, and to be most strongly represented over the occipital electrodes (O1, O2), which we used for subsequent analysis (Wild-Wall et al., 2008; Hsieh and Fang, 2012). These peaks were detected automatically within the time windows 50–150 and 100–200 ms and adaptive mean amplitudes (i.e., the mean amplitude in a 40 ms window centered ±20 ms around the individually detected peak; Clayson et al., 2013) and peak latencies were determined. N2 (200–300 ms) was analyzed at electrode Fz. Amplitudes were measured as peak-to-peak amplitude, relative to the preceding P2 component (Kamijo et al., 2012). This procedure was selected, as the N2 is partially overlapping with preceding P2 and proceeding P3 components and absolute amplitudes can be positive. The P3 (300–600 ms) was analyzed at electrodes Fz and Pz, and adaptive mean amplitudes (±20 ms) were analyzed.

### Statistical Analysis

Statistical analyses were done with SPSS for Windows version 22.0 (IBM Corp., Armonk, NY, United States). Behavioral performance (RT, accuracy, *q*-score) was analyzed with mixed design ANOVAs with the between group factor Age Group (children, young, early middle-aged, late middle-aged, older < 75 years, older > 75 years) and within group factor condition (C, IC). For ERP analyses, the additional factor electrode (O1, O2 for P1 and N1; and Fz, Pz for P3) was added to the ANOVA model. Interaction effects of interest were followed up with pairwise *t*-tests with Bonferroni correction; the corrected *p*-values are reported. Effect sizes were given as partial eta squares (${\text{η}}_{\text{p}}^{2}$). Huynh–Feldt corrections were used when appropriate. In addition, curvilinear regression analyses were performed to assess the influence of age on behavior and ERPs. We further performed an explorative factorial analysis on all ERP parameters measured in the IC conditions (see Table 2 for an overview about the input parameters). Specifically, we employed principle axis factoring as an extraction method (DiStefano et al., 2009) and oblimin as the rotation method in order to appropriately take account for correlations between factors (Costello and Osborne, 2005). We then used regression analysis with the factor scores as regressors for behavioral performance in the IC condition. Only significant (*p <* 0.05) findings are reported.

**Table 2:** Pattern matrix displaying the results of the factor analysis.

	Factor			
	**1**	**2**	**3**	**4**

	**Visual encoding**	**Visual attention**	**Cognitive control**	**Cognitive processing speed**
N1 Latency at O2	**0.930**			
P1 Latency at O1	**0.915**			
N1 Latency at O1	**0.895**			
P1 Latency at O2	**0.882**			
P1 Amplitude at O1	**0.846**			
P1 Amplitude at O2	**0.828**			
P3 Amplitude at Pz	0.463			
N1 Amplitude at O1		**0.921**		
N1 Amplitude at O2		**0.920**		
N2 Amplitude at Fz			**0.654**	
P3 Amplitude at Fz			**0.517**	
N2 Latency at Fz				
P3 Latency at Pz				**0.701**
P3 Latency at Fz				**0.579**

*Bold numbers indicate which variables were summarized within the factor score. Factor loadings < 0.35 are suppressed, for clarity. P3 amplitude at Pz was not included in any of the scores, but treated separately. N2 latencies did not substantially load on any of the factors. All ERP markers included into the factor analysis were obtained from the IC condition.*

## Results

### Behavioral Results

**FIGURE 1:**
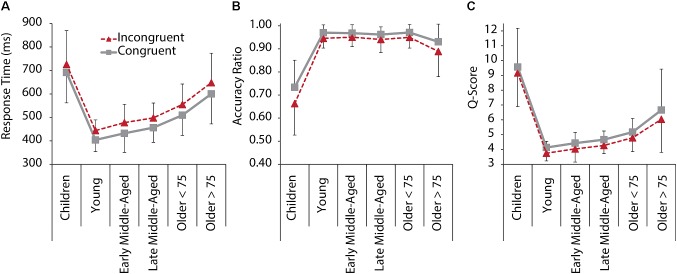
Behavioral performance in the Flanker task for C (gray, solid) and IC (red, dashed) conditions, and all age groups. **(A)** RT. **(B)** Response accuracy ratio. **(C)** *Q*-Score as a standardized performance index, based on reaction time and response accuracy measures per condition. A lower *q*-score represents faster, more accurate performance. Error bars represent standard deviations.

Figure 1 depicts RT, accuracy, and *q*-scores for C and IC trials for all age groups. The 6 Age Group × 2 Condition ANOVA revealed that behavioral performance in IC trials was lower than in C trials [main effects of Condition for RT: *F*(1,205) = 387.105, *p <* 0.001, ${\text{η}}_{\text{p}}^{2}$= 0.654; Accuracy: *F*(1,205) = 75.590, *p <* 0.001, ${\text{η}}_{\text{p}}^{2}$ = 0.269; and *q*-score: *F*(1,205) = 52.090, *p <* 0.001, ${\text{η}}_{\text{p}}^{2}$ = 0.203]. A main effect of Age Group was found for RT, *F*(1,205) = 47.242, *p <* 0.001, ${\text{η}}_{\text{p}}^{2}$ = 0.535, accuracy, *F*(1,205) = 82.077, *p <* 0.001, ${\text{η}}_{\text{p}}^{2}$ = 0.667, and *q*-scores, *F*(1,205) = 63.983, *p* < 0.001, ${\text{η}}_{\text{p}}^{2}$ = 0.609. *Post hoc* comparisons between age groups indicated significantly slower RT in children and older adults > 75 years compared to all other age groups (all *p* < 0.002) and slower RT in older adults < 75 years, compared to young and middle-aged adults (all *p* < 0.001), confirming faster responses with maturation, and slowing of responses with older age. Interestingly, also the two groups of older adults differed significantly from each other, with the older adults > 75 years responding on an even slower level than older < 75 years (*p* = 0.001). With regard to accuracy, children performed on a significantly lower level than all other groups (all *p* < 0.001). Older adults > 75 years had reduced accuracy compared to older adults < 75 years (*p* = 0.039). Young to older adults < 75 years did not differ with regard to response accuracy. Overall task performance as a measure of speed and accuracy (*q*-scores) was lower in children and older adults > 75 years than in the other groups (all *p* < 0.001), but older adults > 75 years still had a significant better performance than children did (Figure 1). Together this suggests that older adults maintain a high level of accuracy while compromising speed.

We also revealed an Age Group × Condition interaction effects for accuracy [*F*(1,205) = 6.015, *p* < 0.001, ${\text{η}}_{\text{p}}^{2}$ = 0.128], suggesting that interference effects differed between age groups. *Post hoc* tests confirmed interference (i.e., reduced accuracy for IC compared to C trials), for all groups, but early middle-aged adults, and interference seemed to be increased in children and older adults > 75 years (Figure 1B).

The lower performance and enhanced interference in children and older adults suggest a curvilinear relationship between age and performance. In order to further test this assumption, we additionally calculated stepwise curvilinear regressions, including age and age-squared as predictors for performance in the IC condition, and confirmed a quadratic relationship, with significant model improvement from linear to quadratic, for all three performance measures (all *p* < 0.001, for quadratic regressions models; see Supplementary Table 1 for regression and change statistics).

In sum, behavioral performance across age groups followed a skewed u-shape pattern, with children and older adults performing on a lower level than young and middle-aged adults, but with older adults still performing better than children do. Moreover, interference effects between groups differed in a way that high performance, as in young and in middle-aged adults, was associated with RT interference effect, while accuracy was not compromised. In children and older adults, however, accuracy was affected by interference.

### Electroencephalographic Results

#### P1 and N1 ERP Components Indexing Visual Processing

**FIGURE 2:**
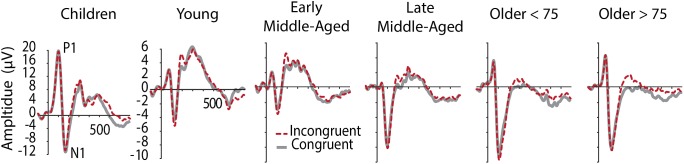
Grand averaged ERPs at occipital electrodes (averaged across O1 and O2), showing visual components P1 and N1 in C (gray, solid) and IC (red, dashed) conditions. Note the different *y*-aches scaling for children.

**FIGURE 3:**
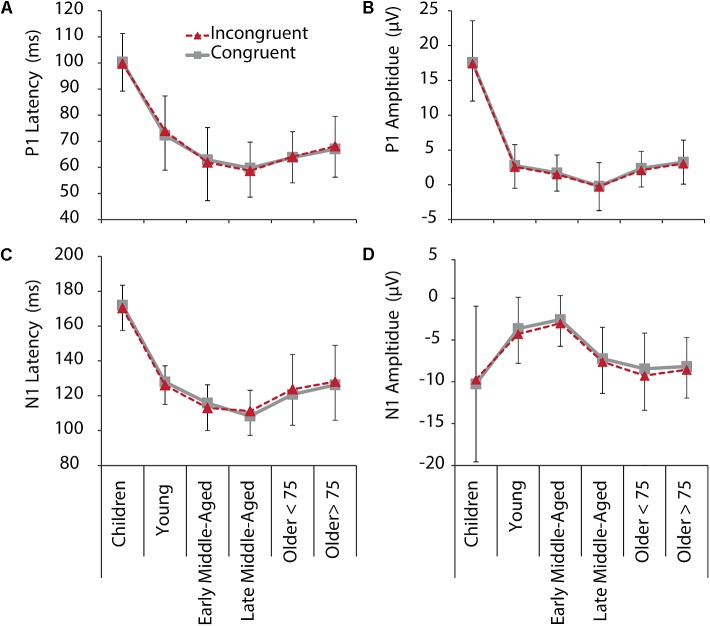
P1 (upper row) and N1 (lower row) latencies and amplitudes for IC (red, dashed) and C (gray, solid) conditions, and all age groups. **(A)** P1 latency. **(B)** P1 amplitude. **(C)** N1 latency. **(D)** N1 amplitude. Negative error bars represent standard deviations for C, while positive error bars represent standard deviations for IC conditions.

Figure 2 shows grand average ERPs averaged across electrodes O1 and O2 for C and IC trials and Figure 3 depicts average latencies and amplitudes for P1 and N1 components for all age groups. By use of a 2 Electrode (O1, O2) × 2 Condition × 6 Age Group ANOVA, we analyzed the visual P1 and N1 components as indexes of visual processing.

For *P1* latency, *F*(5,205) = 87.345, *p* < 0.001, ${\text{η}}_{\text{p}}^{2}$ = 0.681, and amplitude, *F*(5,205) = 145.295, *p* < 0.001, ${\text{η}}_{\text{p}}^{2}$ = 0.780, significant main effects of Age Group were revealed. *Post hoc* tests further showed that children had longer P1 latencies and larger P1 amplitudes, compared to all other age groups (*p* < 0.001, see Figure 2 and note different *y*-axis scaling for children). Latencies further reduced with age from young to middle-aged adults, and were increased again in older adults > 75 years (Figure 3). Throughout adult age amplitudes followed a similar pattern and were larger in young than late middle-aged adults (*p* = 0.019), as well as larger in older adults > 75 years than in late middle-aged adults (*p* = 0.003). Other main and interaction effects were not significant.

Also, for *N1* latency a main effect of Age Group, *F*(5,205) = 93.403, *p* < 0.001, ${\text{η}}_{\text{p}}^{2}$ = 9.695, was found, following the same pattern as P1 latencies, with shortest latencies for middle-aged adults (children > all other groups, young > middle aged, older > middle aged). A main effect of Electrode further indicated overall longer latencies over the left compared to the right hemisphere [*F*(5,205) = 5.113, *p* = 0.025, ${\text{η}}_{\text{p}}^{2}$ = 0.024)]. A Condition by Age Group interaction, *F*(5,205) = 2.969, *p* = 0.013, ${\text{η}}_{\text{p}}^{2}$ = 0.068, suggests different interference effects between groups for N1 latencies. Differences between conditions, however, only were significant in older adults < 75 years (*p* = 0.028). They had longer N1 latencies for IC than C conditions (Figure 3), which might be related to stronger sensory gating for the target color, when only one color is presented.

Also, for N1 amplitudes we confirmed a main effect of Age Group, *F*(5,205) = 9.677, *p* < 0.001, ${\text{η}}_{\text{p}}^{2}$ = 0.191. Again, following a u-shape function, children and older adults had larger N1 amplitudes than young and early middle-aged adults did (all *p* < 0.05). Interestingly, here also late middle-aged adults’ amplitudes, which were indifferent to early middle-aged adults in the other measures, were more similar to older adults (Figure 3), suggesting that N1 amplitudes might be an early marker of age-related changes. Interaction effects for N1 amplitudes were not revealed.

Again, we employed additional curvilinear regression analysis for the IC condition (averaged across electrodes) and confirmed a quadratic relation between age and P1 and N1 amplitudes and latencies (all *p* < 0.001, for quadratic regressions models, see Supplementary Table 2 for regression and change statistics).

#### N2 Components Indexing Interference Suppression

**FIGURE 4:**
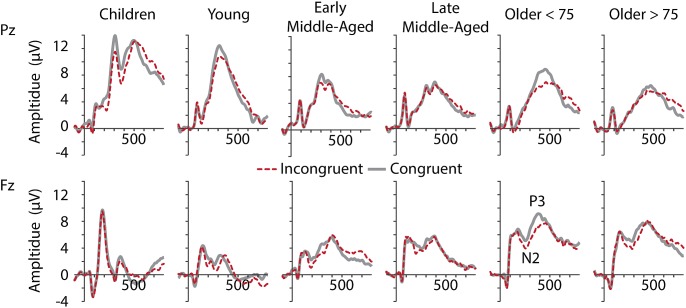
Grand averaged ERPs at electrodes Pz (upper row) and Fz (lower row) for all age groups (from left to right).

**FIGURE 5:**
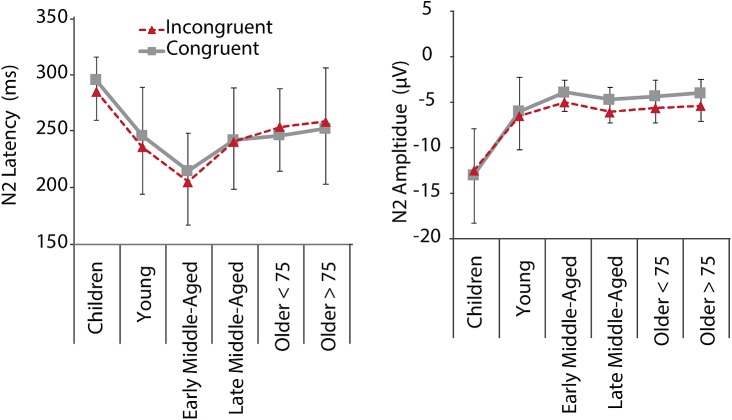
N2 mean latencies and amplitudes at electrode Fz for C (gray, solid) and IC (red, dashed) conditions per age group. Negative error bars represent standard deviations for C, while positive error bars represent standard deviations for IC conditions.

Inspection of grand average data indicated that the N2 component was not a truly negative potential, with amplitudes smaller than zero, but a reduction in amplitude between the preceding P2 and succeeding P3 (see Figure 4, lower panel). We therefore measured N2 amplitudes as peak-to-peak amplitude with respect to the preceding P2 peak. As illustrated in Figure 4, 5, along with latency effects in P1 and N1, N2 latencies were longer in children and older adults [main effect of age: *F*(5,205) = 18.306, *p* < 0.001, ${\text{η}}_{\text{p}}^{2}$ = 0.309]. We confirmed a main effect of Age Group, also for N2 amplitudes, *F*(5,205) = 40.384, *p* < 0.001, ${\text{η}}_{\text{p}}^{2}$ = 0.496. *Post hoc* tests further indicated that children had larger N2 amplitudes than adults (all *p* < 0.001), but no differences between adult age groups. For N2 amplitudes, we further found a main effect of Conditions, *F*(1,205) = 11.749, *p* = 0.001, ${\text{η}}_{\text{p}}^{2}$ = 0.054, and a marginally significant interaction of Conditions and Age. This interaction suggests that differences between the conditions were more pronounced in middle-aged and older adults than in children and young adults (see also Figure 5).

#### P3 ERP Component Indexing Cognitive Updating and Stimulus Categorization

**FIGURE 6:**
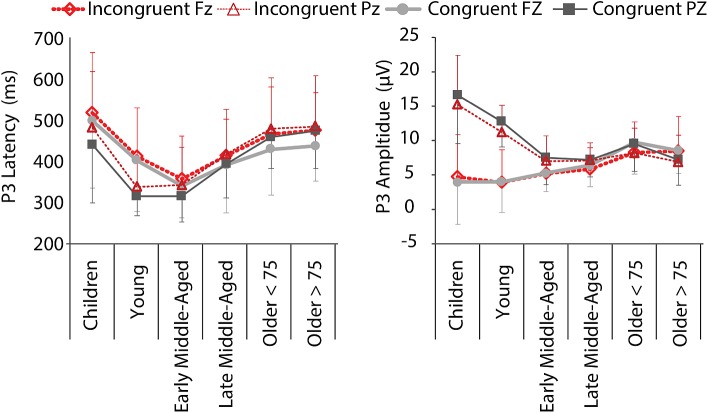
P3 mean latencies and amplitudes at electrodes Fz (thick lines, light colors) and Pz (thin lines, dark colors) for C (gray, solid) and IC (red, dashed) conditions, per age group. Negative error bars represent standard deviations for C, while positive error bars represent standard deviations for IC condition.

We found significant main effects for all factors and both, amplitude and latency [P3 Latency: Electrode, *F*(5,205) = 4.456, *p* = 0.036, ${\text{η}}_{\text{p}}^{2}$ = 0.021; Age Group, *F*(5,205) = 17.273, *p* < 0.001, ${\text{η}}_{\text{p}}^{2}$ = 0. 296; Condition, *F*(5,205) = 19.882, *p* < 0.001, ${\text{η}}_{\text{p}}^{2}$ = 0.088; P3 Amplitude: Electrode, *F*(5,205) = 81.618, *p* < 0.001, ${\text{η}}_{\text{p}}^{2}$ = 0.285; Age Group, *F*(5,205) = 6.454, *p* < 0.001, ${\text{η}}_{\text{p}}^{2}$ = 0.136; Condition, *F*(5,205) = 5.866, *p* = 0.016, ${\text{η}}_{\text{p}}^{2}$ = 0.028; Figure 6], as well as an Electrode by Condition interaction for P3 amplitude, *F*(5,205) = 5.404, *p* = 0.021, ${\text{η}}_{\text{p}}^{2}$ = 0.026. More interestingly, we also revealed Age Group by Electrode interactions for both measures [Latency: *F*(5,205) = 5.745, *p* < 0.001, ${\text{η}}_{\text{p}}^{2}$ = 0.123; Amplitude: *F*(5,205) = 36.665, *p* < 0.000, ${\text{η}}_{\text{p}}^{2}$ = 0.472]. This interaction indicates that P3 latencies were shorter for the parietal electrode than the frontal electrode in children to early middle-aged adults, while the opposite was true in late middle-aged to older adults. Amplitudes were larger at Pz than Fz in children to late middle-aged adults, while the reverse was true for older adults (i.e., P3 was larger at Fz compared to Pz) (Figure 5, 6). This is in agreement with a parietal-to-frontal shift with age, previously reported for this task when comparing young to older adults only (Reuter et al., 2017).

#### Interrelation Between ERPs and Behavioral Performance

In order to investigate the interrelation between behavioral performance and ERPs we performed a regression analysis with ERP parameters measured in the IC condition as predictors for performance in the IC condition (RT, accuracy, *q*-scores) within each age group, as well as across the entire sample. In order to reduce the number of ERP variables, we first ran an explorative factorial analysis on ERP parameters measured in the IC condition prior to the regression analysis. We included amplitudes and latencies for P1 and N1, both at electrodes O1 and O2, N2 at electrode FZ, and P3 at electrodes Fz and Pz in the factor analysis (see Table 2 for an overview about the input parameters).

##### Factorial analysis

Our data were adequate for the factor analysis (KMO = 0.790) and returned four factors explaining a sum of 63.25% of variance (see Table 2 for pattern matrix, showing factor loadings > 0.35). We further calculated the following factor scores based on *z*-standardized variables for subsequent correlation analysis (DiStefano et al., 2009): VISUAL ENCODING (Factor 1: P1 Latencies, P1 Amplitudes, N1 Latencies; note that we did not include P3 amplitude measured at Pz in the calculation of this factor sore, despite loading on this factor); VISUAL ATTENTION (Factor 2: N1 amplitudes); COGNITIVE CONTROL (Factor 3: N2 and P3 amplitude at electrodes Fz); COGNITIVE PROCESSING SPEED (Factor 4: P3 latencies measured at electrodes Fz and Pz). In addition, we included the parietal P3 as individual variable in the regression analysis, deeming it COGNITIVE UPDATING. Analyzing the parietal P3 amplitude separately makes our factors more interpretable from a theoretical point of view and allows us to contrast the parietal and frontal P3 as a marker of age-related changes of brain activation (Reuter et al., 2013, 2017). Note that N2 latency did not load on any of the factors.

##### Regression analysis per age group

We included the four factors gained from factor analysis and P3 amplitude at electrode Pz as predictors for response accuracy, RT, and *q*-scores in the IC Flanker condition in regression models, separately for each group. For RT, this regression model was significant for children, late middle-aged adults, and older adults > 75 years. For accuracy and *q*-scores, the regression model reached significance in children and older adults > 75 years (see Table 3 for model summaries and Supplementary Table 3 for complete coefficient statistics). The factor cognitive processing speed (i.e., P3 latency) was the sole significant predictor for RT (Beta = 0.461, *p* = 0.003) and *q*-scores (Beta = 0.533, *p* = 0.001) in children suggesting that faster cognitive evaluation leads to faster response and ultimately better performance in children. In addition, cognitive control (i.e., frontal N2 and P3 amplitudes. Beta = 0.382, *p* = 0.008) and cognitive updating (i.e., parietal P3, Beta = 0.334, *p* = 0.020) significantly predicted accuracy in children.

**Table 3:** Regression statistics per age group, with factors Visual Encoding, Visual Attention, Cognitive Control, Cognitive Processing Speed, and Cognitive Updating as predictors for RT, accuracy, *q*-scores, and interference effects (i.e., RT and accuracy differences between IC and C conditions).

	Dependent variable	*F*	*df*	Adj *R*^2^	*P*
Children	RT	2.768	5,40	0.164	**0.031**
	Accuracy	2.926		0.176	**0.024**
	*q*-scores	3.158		0.193	**0.017**
	ΔRT	1.533		0.056	0.201
	ΔAccuracy	0.325		-0.081	0.895
Young adults	RT	0.155	5,33	-0.125	0.977
	Accuracy	1.415		0.052	0.244
	*q*-scores	0.310		-0.100	0.904
	ΔRT	2.334		0.149	0.064
	ΔAccuracy	3.022		0.210	**0.024**
Early middle-aged adults	RT	0.558	5,15	-0.124	0.730
	Accuracy	0.535		-0.132	0.747
	*q*-scores	0.525		-0.135	0.754
	ΔRT	0.527		-0.134	0.753
	ΔAccuracy	0.644		-0.098	0.670
Late middle-aged adults	RT	3.177	5,19	0.312	**0.030**
	Accuracy	1.262		0.052	0.320
	q-scores	1.888		0.156	0.144
	ΔRT	1.451		0.086	0.252
	ΔAccuracy	0.711		-0.064	0.623
Older < 75 years	RT	1.882	5,34	0.102	0.129
	Accuracy	0.557		-0.060	0.732
	*q*-scores	2.005		0.114	0.103
	ΔRT	0.354		-0.090	0.876
	ΔAccuracy	0.0531		-0.064	0.751
Older > 75 years	RT	4.320	5,34	0.299	**0.004**
	Accuracy	3.841		0.267	**0.007**
	*q*-scores	3.214		0.221	**0.018**
	ΔRT	0.629		-0.050	0.679
	ΔAccuracy	2.456		0.157	0.053

*Bold writing indicate significant effects at p < 0.05. For models with interference effects (i.e., ΔRT and ΔAccuracy) as dependent variables, factor differences scores were used as independent variables.*

In late middle-aged adults and older < 75 years subjects the factor visual attention (i.e., N1 amplitude) was the sole significant predictor for RT (Beta = 0.561, *p* = 0.009) and *q*-scores (Beta = 0.341 *p* = 0.043), respectively. This suggests that larger visual attention is crucial for fast responses to visual stimuli and better task performance in these age groups. In older adults > 75 years, cognitive updating (i.e., parietal P3) significantly predicted RT (Beta = 0.602, *p* = 0.001), accuracy (Beta = 0.500, *p* = 0.002), and *q*-scores (Beta = 0.500, *p* = 0.002) indicating that a larger, more youth-like parietal P3, is a predictor for faster and more accurate responses. The factor scores were gained from ERP parameters measured in the IC condition. In addition, we also built identical factor scores for the C condition and subtracted those from the IC factor scores in order to obtain difference scores on the factor level. We then performed an additional regression analysis using these difference scores as regressors for interference effects. Only in young adults accuracy interference was significantly predicted by the regression model. Visual encoding difference scores predicted accuracy (Beta = 0.583, *p* = 0.002). This suggests that a relatively reduced increase of visual encoding time for IC compared to C stimuli relates to less accuracy interference. For RT interference, the model was not significant in any of the groups (see Table 3 and Supplementary Table 4 for complete statistics).

##### Regression analysis across the entire sample

In addition to the group-wise analysis, we also conducted a stepwise multiple regression analysis, with age and age-squared (age2) entered in the first step, and the predictors gained from the ERP data in the second step in our entire sample. For RT, accuracy, and *q*-scores, a significant model change was confirmed when the ERP parameters were included (see Supplementary Table 5 for statistics), confirming that the neurophysiological markers have an explanatory value for behavioral performance when age has been already accounted for. In addition to age and age2, cognitive processing speed (i.e., P3 latency, Beta = 0.175, *p* = 0.004) and visual encoding (i.e., P1 and N1 latencies, and P1 amplitude; Beta = 0.294, *p* = 0.006; note that most variables loading on that factors were latencies, and hence this could be interpreted as faster visual encoding relating to faster RT) significantly predicted RT. With regard to accuracy, cognitive processing speed (Beta = 0.106, *p* = 0.037), cognitive updating (i.e., P3 amplitudes, Beta = 0.260, *p* = 0.001), and visual encoding (Beta = 0.446, *p* = 0.001) were identified as significant predictors in addition to age and age2. Similarly, with regard to *q*-scores, cognitive processing speed (Beta = 0.216, *p* < 0.001), cognitive updating (i.e., P3 amplitudes, Beta = 0.173, *p* = 0.004), and visual encoding (Beta = 0.395, *p* < 0.001) were identified as significant predictors. Regression models with difference scores as predictors for interference effects were not significant.

## Discussion

Children and older adults have less effective attentional control associated with incomplete maturation and aging of the brain. Taking a lifespan perspective, here, we aimed (1) to reveal, whether similar behavioral performance deficits in children and older adults are associated with similar or different electrophysiological markers and (2) to learn more about the development of selective attention and its electrophysiological correlates across the middle-age lifespan. We confirmed that behavioral performance across age follows a skewed u-shape function, with peak performance in young adulthood. Notably however, this function seemed larger skewed with children performing on an even lower level than older adults do. Especially, while RT seemed to progressively slow from young adults to older ages, accuracy was largely stable across the adult lifespan and was only reduced in the oldest old (> 75 years of age). Also, EEG markers of encoding and processing speed (i.e., P1, N1, N2, and P3 latencies), as well as markers of visual processing and attention (i.e., P1, N1 amplitudes), followed an u-shape pattern. Notably, these EEG markers peaked in middle adulthood. Markers of cognitive processing (i.e., N2, P3 amplitudes) seemed to change more gradually across the lifespan. Most interestingly, cognitive processing speed predicts RT and overall task performance (*q*-scores); and cognitive control and updating predict response accuracy in children. By contrast, in older adults cognitive updating and stimulus categorization resources seem to be key determinates for all performance outcomes.

### Low Performance in Children and Slow Responses in Older Adults

Children and older adults performed on a lower level than young and middle-aged adults in the colored Flanker task. These findings are in line with previous reports comparing children (e.g., Ridderinkhof and Molen, 1995; Ridderinkhof and van der Stelt, 2000) or older adults (e.g., Wild-Wall et al., 2008; Reuter et al., 2017) with young or middle-aged participants and confirm previous lifespan comparisons (Li et al., 2009; Waszak et al., 2010). Notably, however, even our oldest group, including subjects aged between 76 and 83 years, still performed on a significantly higher level than children (Figure 1C). Specifically, while children performed both less accurate and slower than young and middle-aged participants did, in older adults, mainly speed was compromised, but accuracy was largely maintained. This suggests that inhibitory control is relatively well maintained up to old age, but comes at a cost of slowing. On a descriptive level, RT slowing seems to start already in early middle-aged and then to gradually increase toward old age (Figure 1A). Slowing with age is a well-described phenomenon (Buckles, 1993; Salthouse, 2000; Woods et al., 2015). Previous research suggests that slowing in middle-adulthood is primarily related to changes in the response criterion (i.e., middle-aged adults are less likely to respond quick, but prefer to accumulate more evidence to avoid errors), while older adults are faced with an additional loss of perceptual-motor processing speed and a reduction of attentional resources (Godefroy et al., 2010). The relatively maintained accuracy into older age, the increased slowing of RT with advancing age, and the short peak latencies for all ERP components in middle-aged adults support this notion (Figure 3).

In addition to speed and accuracy, another crucial marker of selective attention is *interference*, i.e., reduced accuracy and prolonged RT for IC compared to C stimuli (Diamond, 2013). We found interference effects for both speed and accuracy (Wild-Wall et al., 2008; Hsieh and Fang, 2012). Interestingly, with regard to RT, this effect was of equal size in all groups. For accuracy, by contrast, interference was larger in children and older adults > 75 years, supporting the notion that inhibitory control is not yet fully developed in children and compromised in older age (for review, see Diamond, 2013).

### ERP Markers Suggest Premium Functioning in Middle-Age

Also with regard to the electrophysiological markers, our results largely confirm previous findings comparing either children or older adults with young adults (for reviews, see e.g., Segalowitz et al., 2010; Pires et al., 2014). P1 and N1 amplitudes followed a u-shape pattern, with larger amplitudes in longer latencies at both ends of the lifespan. Notably, and similarly to the behavioral data, this distribution was strongly skewed (Figure 3). A reduction of intracortical inhibition most likely contributes to enlarged visual evoked ERPs at both ends of the lifespan (Pires et al., 2014). In addition, increase in N1 amplitudes in older adults might be a consequence of increased visual attention devoted to the target stimuli (Wild-Wall et al., 2008). The particular large amplitudes in children could further be linked to structural changes in the gray matter, myelination, dendritic arborization, synaptic pruning, alterations in neurotransmitter levels, and increasing head volume and skull thickness, that are yet to occur (Whitford et al., 2007; Segalowitz et al., 2010; Simmonds et al., 2014). These developmental changes might also account for the larger N2 and parietal P3 amplitudes in children (Casey et al., 2000; Segalowitz et al., 2010). N2 amplitudes did not differ between adult groups. However, the main effect of condition on N2 amplitudes tended to be modulated by age. Specifically, it seemed to be driven by larger N2 amplitudes in the IC condition in late middle-aged to older adults only. By contrast, there were only small differences between conditions in children, young, and early middle-aged adults. This might suggest that from late middle-age to older age, participants engaged relatively more response inhibition processes following IC stimuli. We suggest that children do not yet have sufficient inhibitory capacities to effectively engage response inhibition. This interpretation is in line with the reduced accuracy level in children and literature suggesting that children have a reduced ability to control attentional focus (Johnstone et al., 1996; Mueller et al., 2008; Boucher et al., 2010; Waszak et al., 2010). However, the absence of N2 amplitude modulation in young and early middle-aged adults seems more puzzling. We suggest that the task was sufficiently easy for these groups that they did not need to engage strong response inhibitory processes to perform on a high level.

P3 amplitudes were larger and latencies shorter for C stimuli in all age groups, suggesting developmental stability rather than change with regard to stimulus categorization, updating, and target identification. The P3 distribution, however, showed the expected parietal-to-frontal shift with age (Fabiani et al., 1998; Perchet and Garcia-Larrea, 2005; Davis et al., 2008). Specifically, the P3 had a strong parietal focus in children and in young adults, was equipotent at frontal and parietal electrodes in middle-aged adults, and had a frontal focus in the oldest groups. Latencies of all components were prolonged in children and elderly reflecting slower processing and reduced cognitive efficiency, which is likely to be related to the reduced myelination and lower nerve conduction velocity early and late in life (Casey et al., 2000; Ridderinkhof and van der Stelt, 2000).

More interestingly, when we consider the ERP findings across all age groups, it is striking that peak “performance” (i.e., the reversal point in the u-shape distribution), seemed to be in middle-age and not in young adults (Figure 3, 5). Our young adults had an average age of about 23 years. Brain maturation, however, might not be completed until the mid- to late-20ths (for review, see Somerville, 2016). Thus, despite being at their top behavioral performance level, we suggest that ongoing brain maturation contributes to the delay in the peak ERP “performance” (note however, that our group of young adults did not differ significantly from early middle-aged adults in any of our measure, so that this observation is merely descriptive). Nevertheless, the question arises, how young adults seem to respond faster, while middle age adults process the stimuli quicker. We assume that young adults might have reduced response criterions, and maybe are also faster in motor response generation. This fits to the notion that the motor cortex is matured at the age of 15 years (Huttenlocher, 1979).

### Processes of Growth and Deterioration Are Qualitatively Different

The temporal inconsistencies between peak behavior and peak EEG “performance,” as well as the gradual changes in P3 distribution across age already suggest that the relationship between electroencephalographic measures and performance is not uniform across the lifespan. Furthermore, despite the seemingly mirror-symmetrical patterns in some measures in childhood and senescence, processes of growth and deterioration might be qualitatively different in many respects (Span et al., 2004). In order to learn more about the relation between ERP measures and behavioral performance in the flanker task we conducted linear regression analyses, separately for all age groups. We found that ERP markers significantly predicted performance mainly in children and older adults > 75 years, but also in the late middle-aged sample. The variability between subjects in RTs and accuracy in the other age groups might have been too low due to ceiling effects to show any relations. Nevertheless, the analysis suggests an interesting distinction between children and older adults > 75 years. P3 latencies, indicating cognitive processing and stimulus evaluation speed significantly predicted RT, and *q*-scores in children, while cognitive control (N2 and frontal P3) and updating (parietal P3) predicted accuracy. This suggests that for children, not the delayed perceptual encoding speed, but the speed and resources, related to cognitive stimulus categorization, restrict performance. In older adults, not speed at all, but only the cognitive resources engaged to update and evaluate the stimulus category predicted Flanker performance. RT were faster and accuracy was better when parietal P3 amplitudes were larger, and thus suggesting more youth like processing (for a detailed discussion on P3 distribution and flanker performance in older adults, see Reuter et al., 2017). Together, these results suggest that in children gains in information-processing speed are the key to the improvement in executive function, while in older adults, the (remaining) available cognitive resources determine behavioral outcomes. This contrasts the general-resource account of lifespan development, which attributes age-related differences in cognitive capabilities to the development of information processing speed across the lifespan (Kail and Salthouse, 1994; Waszak et al., 2010). They, however, support early findings by Span et al. (2004), suggesting that only in children processing speed is a general predictor for performance, while in older adults age-effects emerge over and above global-speed effects (Span et al., 2004). In line with our previous findings (Reuter et al., 2017), we did not find support for compensatory recruitment of frontal resources with increasing age in the Flanker task. However, in late middle-adulthood and the oldest old, more visual attention (i.e., larger N1 amplitudes) was associated with shorter RT and better overall performance, respectively. This might be a compensatory process that buffers early age-related losses in processing speed (Wild-Wall et al., 2008).

Differences in between conditions in ERP parameters were not shown to be predictive of behavioral interference effects. Only in young adults a reduced increase of visual encoding time for IC compared to C stimuli relates to less accuracy interference, suggesting that efficient visual encoding is a key requirement for interference suppression in young adults.

Independent of age and across the entire sample, RT and accuracy were predicted by the factors cognitive processing speed (i.e., P3 latencies) and visual encoding (i.e., P1 and N1 latencies). This confirms that, independent of age, perceptual and cognitive processing speed, well relate to cognitive performance. In addition, the factor cognitive updating (i.e., P3 amplitudes) also significantly predicted accuracy. With smaller samples, relationships between ERP markers and performance outcomes often do not emerge. Yet here, our findings highlight the relevance of studying ERP markers to learn about executive functioning.

### Methodological Considerations and Conclusion

We aimed to study selective attention across the lifespan. In particular, we attempted to contrast children with older adults, and to inspect development across the middle-aged lifespan. There are, however, some aspects that we did not consider in the study. First, despite having data from groups of participants across the lifespan, we do not have a continuous sample. In the adult age range, we are only missing subjects aged between 30 and 35 and between 49 and 54 years of age. However, more crucially is the fact that we do not have an adolescent sample. Brain development and improvement in executive function is immense throughout adolescence (Huizinga et al., 2006; Pozuelos et al., 2014). We can assume that RT would progressively decrease while accuracy would increase from childhood to early adulthood; nevertheless, an adolescent sample would have nicely complemented our data set. Another caveat are the ceiling effects in young and middle-aged adults’ performance accuracy. We selected a colored version of the flanker task as it has been shown that children and older adults were able to perform the task at ease (McDermott et al., 2007; Voelcker-Rehage et al., 2010; Waszak et al., 2010). As a consequence, however, the task was very easy for young and middle-aged adults, and even older adults < 75 years (but note that other studies reported similar accuracy rates, e.g., Zeef and Kok, 1993; Colcombe et al., 2005; Salthouse, 2010; Hsieh and Fang, 2012; Hsieh and Lin, 2014; Korsch et al., 2014; Li et al., 2014; Chuang et al., 2015; de Bruin and Della Sala, 2017). A task with an adaptive difficulty level might be an alternative option to overcome this problem in the future. Another issue is that we did not asses simple processing speed and thus we could not control for general slowing, independent of the task. At last, we had an unequal gender distribution in our sample, because for Study 2 only female participants were recruited. This leads to an over-representation of females in our young and the two older samples. Previous studies report prolonged RT and reduced accuracy in females (e.g., Clayson et al., 2011; Larson et al., 2011) and increased interference effects (e.g., Stoet, 2010; Judge and Taylor, 2012; Fischer et al., 2016). Therefore, it could be possible that the over-representation of female participants in our older samples has contributed to the seemingly steeper performance decrease in older age. Notably, however, if we re-run all ANOVA models separately for both genders, largely identical results are revealed for both groups. The only exception is that the interaction between condition and age group for N1 amplitudes only emerged in the analysis for females, which is in line with the notion that females might be more susceptible by flankers than male participants. Given the general similarity of results for males and females, we are, however, confident that the pattern of results is reliable independent of genders.

To conclude, we confirmed the (skewed) u-shape distribution for behavioral performance and some electrophysiological markers (latencies of all components, and P1 and N1 amplitudes) across the lifespan. We further revealed important distinctions between children and older adults: (1) children and older adults are not mirroring, but children perform on a much lower level with both speed and accuracy being compromised, and children have more extreme ERP parameters and (2) children’s performance depends on speed of information processing (i.e., P3 latencies) while older adults performance depends on the cognitive resources used to evaluate the stimulus (i.e., parietal P3 amplitude). We also found that peak behavioral performance seems to be in young adults, but peak speed of information processing (i.e., ERP latencies) and peak deployment of visual attention (N1 amplitudes) in middle age. Thus, the age-related decline starts later than most young–old comparison suggest. Together, this highlights the benefit of including middle-aged samples when investigating age-related differences, as it suggests that the changes from young adulthood to old age are not unidirectional. In sum, different mechanisms seem to restrict performance early and late in life and suggest a non-linear relationship between electrophysiological markers and performance in the Flanker task across the lifespan.

## Author Contributions

All authors contributed to the design of the study, offered comments on the manuscript, and approved the final version. E-MR, SV, FK, and LH collected the data. E-MR analyzed the data, wrote the initial draft of the manuscript, and performed the revision of the manuscript. E-MR, SV, CV-R, HB, and BG discussed and interpreted the results and discussed the outline of the manuscript.

## Conflict of Interest Statement

The authors declare that the research was conducted in the absence of any commercial or financial relationships that could be construed as a potential conflict of interest.
