# Supplementary material for: A Non-linear Relationship Between Selective Attention and Associated ERP Markers Across the Lifespan
Source: Front Psychol. 2019 Jan 28;10:30. doi: 10.3389/fpsyg.2019.00030 (PMC6360996; doi:10.3389/fpsyg.2019.00030)
Supplement: Supplementary file 1 [file Data_Sheet_1.docx]

# Supplementary Material

**A non-linear relationship between selective attention and associated ERP markers across the lifespan**

Running Head: Selective attention and associated ERP markers across the lifespan

Eva-Maria Reuter*^1^, Solveig Vieluf^2^, Flora Koutsandreou^3^, Lena Hübner^4^, Henning Budde^3,5,6^, Ben Godde^7^, Claudia Voelcker-Rehage^4^

^1^Centre for Sensorimotor Performance, School of Human Movement and Nutrition Sciences, The University of Queensland, Brisbane, QLD, Australia

^2^Institute of Sports Medicine, University of Paderborn, Paderborn, Germany

^3^Faculty of Human Sciences, Medical School Hamburg, Hamburg, Germany

^4^Institute of Human Movement Science and Health, Chemnitz University of Technology, Chemnitz, Germany

^5^Physical Activity, Physical Education, Health and Sport Research Centre (PAPESH), Sports Science Department, School of Science and Engineering, Reykjavik University, Reykjavik, Iceland

^6^Lithuanian Sports University, Kaunas, Lithuania

^7^Psychology and Methods, Jacobs University, Bremen, Germany

*Correspondence to this article should be directed to:

Eva-Maria Reuter

School of Human Movement and Nutrition Sciences

The University of Queensland

Brisbane, QLD 4072

email: e.reuter@uq.edu.au

phone: (+61 7) 3365 6104

**Supplementary Table 1.** Statistic results of curvilinear regression analysis with age and age-squared (age2) as a predictor for behavioural performance in the incongruent condition

|  | Regression Statistics | | | | |  | | | |
| --- | --- | --- | --- | --- | --- | --- | --- | --- | --- |
|  | Model | R Square | Adjusted R Square | Std. Error of the Estimate | R Square Change | F Change | Df1 | Df2 | Sig. F Change |
| RT | |  |  |  |  |  |  |  |  |
|  | Age | .107 | .007 | 145.145 | .012 | 2.402 | 1 | 206 | .123 |
|  | Age2 | .643 | .408 | 112.054 | .402 | 140.638 | 1 | 205 | .000 |
| Accuracy | |  |  |  |  |  |  |  |  |
|  | Age | .243 | .239 | 0.125 | .243 | 66.2178 | 1 | 206 | .000 |
|  | Age2 | .528 | .523 | 0.099 | .285. | 123.737 | 1 | 205 | .000 |
| Q-Score | |  |  |  |  |  |  |  |  |
|  | Age | .093 | .089 | 2.590 | .093 | 21.135 | 1 | 206 | .000 |
|  | Age2 | .457 | .451 | 2.010 | .364 | 137.258 | 1 | 205 | .000 |

**Supplementary Table 2.** Statistic results of curvilinear regression analysis with age as a predictor for P1 and N1 latencies and amplitudes in the incongruent condition.

|  | Regression Statistics | | | | |  | | | |
| --- | --- | --- | --- | --- | --- | --- | --- | --- | --- |
|  | Model | R Square | Adjusted R Square | Std. Error of the Estimate | R Square Change | F Change | Df1 | Df2 | Sig. F Change |
| P1 Latency | |  |  |  |  |  |  |  |  |
|  | Age | .413 | .410 | 13.834 | .413 | 145.139 | 1 | 206 | .000 |
|  | Age2 | .650 | .646 | 10.713 | .237 | 138.523 | 1 | 205 | .000 |
| P1 Amplitude | |  |  |  |  |  |  |  |  |
|  | Age | .374 | .371 | 5.897 | .374 | 12.979 | 1 | 206 | .000 |
|  | Age2 | .672 | .669 | 4.275 | .229 | 186.908 | 1 | 205 | .000 |
| N1 Latency | |  |  |  |  |  |  |  |  |
|  | Age | .287 | .284 | 21.666 | .287 | 83.077 | 1 | 206 | .000 |
|  | Age2 | .618 | .614 | 15.907 | .330 | 117.136 | 1 | 205 | .000 |
| N1 Amplitude | |  |  |  |  |  |  |  |  |
|  | Age | .005 | .000 | 5.804 | .005 | 0.941 | 1 | 206 | .000 |
|  | Age2 | .101 | .092 | 5.529 | .096 | 21.992 | 1 | 205 | .000 |

**Supplementary Table 3.** Coefficient statistics per age group for regression analysis with factors Visual encoding, Visual attention, Cognitive control, Cognitive Processing Speed and Cognitive Updating as predictors for RT, accuracy, and q-scores.

| Age group | Predictors | Reaction time | | |  | Accuracy | | |  | Q-scores | | |
| --- | --- | --- | --- | --- | --- | --- | --- | --- | --- | --- | --- | --- |
|  |  | β | t | p |  | β | t | p |  | β | t | p |
| Children | Constant |  | 7.535 | 0.000 |  |  | 8.340 | 0.000 |  |  | 4.935 | 0.000 |
|  | Cognitive Processing Speed | 0.461 | 3.163 | 0.003 |  | -0.104 | -0.717 | 0.477 |  | 0.533 | 3.717 | 0.001 |
|  | Cognitive Control | -0.119 | -0.869 | 0.390 |  | -0.382 | -2.815 | 0.008 |  | 0.174 | 1.299 | 0.202 |
|  | Visual Attention | -0.137 | -0.963 | 0.341 |  | 0.017 | 0.119 | 0.906 |  | -0.129 | -0.926 | 0.360 |
|  | Visual Encoding | 0.011 | 0.081 | 0.936 |  | -0.165 | -1.167 | 0.250 |  | 0.164 | 1.172 | 0.248 |
|  | Cognitive Updating | 0.147 | 1.063 | 0.294 |  | 0.334 | 2.429 | 0.020 |  | -0.076 | -0.560 | 0.579 |
| Young | Constant |  | 24.621 | 0.000 |  |  | 58.686 | 0.000 |  |  | 25.225 | 0.000 |
|  | Cognitive Processing Speed | 0.060 | 0.314 | 0.756 |  | -0.088 | -0.501 | 0.620 |  | 0.094 | 0.498 | 0.622 |
|  | Cognitive Control | 0.150 | 0.700 | 0.489 |  | 0.182 | 0.927 | 0.360 |  | 0.071 | 0.334 | 0.740 |
|  | Visual Attention | -0.096 | -0.452 | 0.654 |  | -0.084 | -0.429 | 0.671 |  | -0.057 | -0.271 | 0.788 |
|  | Visual Encoding | 0.044 | 0.212 | 0.833 |  | -0.247 | -1.286 | 0.207 |  | 0.146 | 0.707 | 0.484 |
|  | Cognitive Updating | -0.105 | -0.590 | 0.559 |  | 0.193 | 1.176 | 0.248 |  | -0.181 | -1.028 | 0.312 |
| Early middle-aged | Constant |  | 9.884 | 0.000 |  |  | 32.347 | 0.000 |  |  | 9.725 | 0.000 |
|  | Cognitive Processing Speed | 0.273 | 1.132 | 0.275 |  | 0.170 | 0.702 | 0.493 |  | 0.223 | 0.922 | 0.371 |
|  | Cognitive Control | -0.009 | -0.039 | 0.970 |  | -0.211 | -0.860 | 0.403 |  | 0.038 | 0.155 | 0.879 |
|  | Visual Attention | -0.113 | -0.389 | 0.703 |  | 0.313 | 1.074 | 0.300 |  | -0.179 | -0.615 | 0.548 |
|  | Visual Encoding | 0.355 | 1.403 | 0.181 |  | -0.081 | -0.318 | 0.755 |  | 0.365 | 1.434 | 0.172 |
|  | Cognitive Updating | -0.049 | -0.175 | 0.864 |  | 0.338 | 1.197 | 0.250 |  | -0.129 | -0.455 | 0.655 |
| Late middle-age | Constant |  | 14.696 | 0.000 |  |  | 26.159 | 0.000 |  |  | 13.132 | 0.000 |
|  | Cognitive Processing Speed | 0.420 | 1.903 | 0.072 |  | 0.142 | 0.549 | 0.589 |  | 0.339 | 1.389 | 0.181 |
|  | Cognitive Control | 0.144 | 0.708 | 0.487 |  | 0.235 | 0.987 | 0.336 |  | 0.019 | 0.084 | 0.934 |
|  | Visual Attention | 0.561 | 2.919 | 0.009 |  | 0.311 | 1.380 | 0.184 |  | 0.416 | 1.953 | 0.066 |
|  | Visual Encoding | -0.078 | -0.367 | 0.718 |  | -0.031 | -0.122 | 0.904 |  | -0.052 | -0.221 | 0.827 |
|  | Cognitive Updating | 0.119 | 0.569 | 0.576 |  | 0.342 | 1.393 | 0.180 |  | -0.062 | -0.269 | 0.791 |
| Older <75 | Constant |  | 22.418 | 0.000 |  |  | 68.865 | 0.000 |  |  | 20.009 | 0.000 |
|  | Cognitive Processing Speed | -0.188 | -1.155 | 0.256 |  | 0.042 | 0.235 | 0.815 |  | -0.198 | -1.226 | 0.229 |
|  | Cognitive Control | 0.058 | 0.355 | 0.725 |  | 0.012 | 0.065 | 0.949 |  | 0.044 | 0.272 | 0.787 |
|  | Visual Attention | 0.287 | 1.746 | 0.090 |  | -0.271 | -1.517 | 0.139 |  | 0.349 | 2.137 | 0.040 |
|  | Visual Encoding | -0.229 | -1.296 | 0.204 |  | 0.005 | 0.025 | 0.980 |  | -0.251 | -1.425 | 0.163 |
|  | Cognitive Updating | -0.282 | -1.736 | 0.092 |  | 0.068 | 0.386 | 0.702 |  | -0.245 | -1.519 | 0.138 |
| Older>75 | Constant |  | 19.200 | 0.000 |  |  | 34.427 | 0.000 |  |  | 8.020 | 0.000 |
|  | Cognitive Processing Speed | 0.107 | 0.724 | 0.474 |  | -0.140 | -0.927 | 0.360 |  | 0.150 | 0.964 | 0.342 |
|  | Cognitive Control | 0.174 | 1.287 | 0.207 |  | -0.127 | -0.919 | 0.365 |  | 0.120 | 0.844 | 0.404 |
|  | Visual Attention | 0.182 | 1.284 | 0.208 |  | -0.188 | -1.297 | 0.203 |  | 0.182 | 1.221 | 0.231 |
|  | Visual Encoding | -0.002 | -0.017 | 0.987 |  | -0.209 | -1.398 | 0.171 |  | 0.144 | 0.935 | 0.357 |
|  | Cognitive Updating | -0.602 | -4.197 | 0.000 |  | 0.500 | 3.416 | 0.002 |  | -0.500 | -3.311 | 0.002 |

**Supplementary Table 4.** Coefficient statistics per age group for regression analysis with factor difference scores (incongruent – congruent conditions) as predictors for behavioural interference.

| Age group | Predictors | Reaction time Interference | | |  | Accuracy Interference | | |  |
| --- | --- | --- | --- | --- | --- | --- | --- | --- | --- |
|  |  | β | t | p |  | β | t | p |  |
| Children | Constant |  | 4.721 | 0.000 |  |  | -5.472 | 0.000 |  |
|  | ΔCognitive Processing Speed | 0.293 | 1.865 | 0.069 |  | 0.007 | 0.042 | 0.967 |  |
|  | ΔCognitive Control | 0.061 | 0.400 | 0.691 |  | 0.043 | 0.264 | 0.793 |  |
|  | ΔVisual Encoding | -0.215 | -1.333 | 0.190 |  | 0.141 | 0.814 | 0.421 |  |
|  | ΔCognitive Updating | 0.121 | 0.718 | 0.477 |  | 0.011 | 0.062 | 0.951 |  |
|  | ΔVisual Attention Interference | -0.001 | -0.006 | 0.995 |  | -0.179 | -1.053 | 0.298 |  |
| Young | Constant |  | 12.018 | 0.000 |  |  | -4.286 | 0.000 |  |
|  | ΔCognitive Processing Speed | 0.406 | 2.468 | 0.019 |  | 0.046 | 0.291 | 0.773 |  |
|  | ΔCognitive Control | 0.112 | 0.687 | 0.497 |  | 0.131 | 0.831 | 0.412 |  |
|  | ΔVisual Encoding | 0.011 | 0.060 | 0.953 |  | -0.583 | -3.286 | 0.002 |  |
|  | ΔCognitive Updating | -0.275 | -1.656 | 0.107 |  | 0.025 | 0.157 | 0.876 |  |
|  | ΔVisual Attention Interference | 0.168 | 0.893 | 0.378 |  | 0.291 | 1.605 | 0.118 |  |
| Early middle-aged | Constant |  | 9.508 | 0.000 |  |  | -1.522 | 0.149 |  |
|  | ΔCognitive Processing Speed | 0.163 | 0.608 | 0.552 |  | 0.328 | 1.245 | 0.232 |  |
|  | ΔCognitive Control | -0.005 | -0.016 | 0.987 |  | 0.036 | 0.128 | 0.900 |  |
|  | ΔVisual Encoding | 0.086 | 0.343 | 0.736 |  | -0.064 | -0.262 | 0.797 |  |
|  | ΔCognitive Updating | -0.297 | -0.910 | 0.377 |  | 0.133 | 0.413 | 0.685 |  |
|  | ΔVisual Attention Interference | -0.215 | -0.835 | 0.417 |  | -0.124 | -0.489 | 0.632 |  |
| Late middle-age | Constant |  | 8.782 | 0.000 |  |  | -1.875 | 0.076 |  |
|  | ΔCognitive Processing Speed | 0.264 | 1.226 | 0.235 |  | 0.291 | 1.251 | 0.226 |  |
|  | ΔCognitive Control | -0.135 | -0.674 | 0.509 |  | 0.206 | 0.953 | 0.353 |  |
|  | ΔVisual Encoding | 0.062 | 0.275 | 0.786 |  | 0.177 | 0.731 | 0.474 |  |
|  | ΔCognitive Updating | -0.404 | -2.012 | 0.059 |  | -0.032 | -0.147 | 0.884 |  |
|  | ΔVisual Attention Interference | -0.097 | -0.427 | 0.674 |  | -0.227 | -0.922 | 0.368 |  |
| Older <75 | Constant |  | 8.757 | 0.000 |  |  | -3.306 | 0.002 |  |
|  | ΔCognitive Processing Speed | -0.057 | -0.334 | 0.741 |  | -0.202 | -1.203 | 0.237 |  |
|  | ΔCognitive Control | -0.083 | -0.438 | 0.664 |  | -0.035 | -0.187 | 0.853 |  |
|  | ΔVisual Encoding | 0.026 | 0.149 | 0.883 |  | 0.089 | 0.512 | 0.612 |  |
|  | ΔCognitive Updating | -0.163 | -0.884 | 0.383 |  | -0.131 | -0.716 | 0.479 |  |
|  | ΔVisual Attention Interference | 0.098 | 0.544 | 0.590 |  | 0.042 | 0.237 | 0.814 |  |
| Older>75 | Constant |  | 9.623 | 0.000 |  |  | -5.049 | 0.000 |  |
|  | ΔCognitive Processing Speed | -0.079 | -0.446 | 0.659 |  | -0.014 | -0.087 | 0.931 |  |
|  | ΔCognitive Control | 0.102 | 0.491 | 0.626 |  | -0.260 | -1.400 | 0.171 |  |
|  | ΔVisual Encoding | 0.091 | 0.554 | 0.583 |  | -0.441 | -2.994 | 0.005 |  |
|  | ΔCognitive Updating | -0.206 | -1.056 | 0.299 |  | 0.278 | 1.590 | 0.121 |  |
|  | ΔVisual Attention Interference | 0.179 | 1.063 | 0.295 |  | -0.071 | -0.473 | 0.639 |  |

**Supplementary Table 5:** Statistic results of stepwise regression analysis with age and age-squared (age2), as well as factors based on ERP markers as predictors for behavioural performance in the incongruent condition (RT, Accuracy and Q-scores) as well as for interference effects (ΔRT, ΔAccuracy).

|  | Regression Statistics | | | | |  | | | |
| --- | --- | --- | --- | --- | --- | --- | --- | --- | --- |
|  | Model | R Square | Adjusted R Square | Std. Error of the Estimate | R Square Change | F Change | Df1 | Df2 | Sig. F Change |
| RT | |  |  |  |  |  |  |  |  |
|  | Age Age2 | .414 | .408 | 112.05350 | .414 | 72.334 | 2 | 205 | .000 |
|  | Visual encoding  Visual attention  Cognitive control  Cognitive Processing Speed | .464 | .446 | 108.42937 | .051 | 3.787 | 5 | 200 | .003 |
| Accuracy | |  |  |  |  |  |  |  |  |
|  | Age Age2 | .528 | .523 | .09925 | .528 | 114.672 | 2 | 205 | .000 |
|  | Visual encoding  Visual attention  Cognitive control  Cognitive Processing Speed | .625 | .612 | .08957 | .097 | 10.333 | 5 | 200 | .000 |
| Q-score | |  |  |  |  |  |  |  |  |
|  | Age Age2 | .457 | .451 | 2.00950 | .457 | 86.186 | 2 | 205 | .000 |
|  | Visual encoding  Visual attention  Cognitive control  Cognitive Processing Speed | .545 | .529 | 1.86244 | .088 | 7.730 | 5 | 200 | .000 |
| ΔRT |  |  |  |  |  |  |  |  |  |
|  | Age Age2 | .019 | .009 | 29.90554 | .019 | 1.951 | 2 | 205 | .145 |
|  | ΔVisual encoding  ΔVisual attention  ΔCognitive control  ΔCognitive ΔProcessing Speed | .055 | .022 | 29.70747 | .037 | 1.549 | 5 | 200 | .176 |
| ΔAccuracy |  |  |  |  |  |  |  |  |  |
|  | Age Age2 | .113 | .104 | .05306 | .113 | 13.019 | 2 | 205 | .000 |
|  | ΔVisual encoding  ΔVisual attention  ΔCognitive control  ΔCognitive ΔProcessing Speed | .136 | .106 | .05300 | .023 | 1.087 | 5 | 200 | .369 |
